# Supplementary material for: A guanidine-degrading enzyme controls genomic stability of ethylene-producing cyanobacteria
Source: Nat Commun. 2021 Aug 26;12:5150. doi: 10.1038/s41467-021-25369-x (PMC8390497; doi:10.1038/s41467-021-25369-x)
Supplement: Supplementary file 1 — Supplementary Information [file 41467_2021_25369_MOESM1_ESM.pdf]

A guanidine-degrading enzyme controls genomic stability of ethylene-producing cyanobacteria

Wang *et al*.

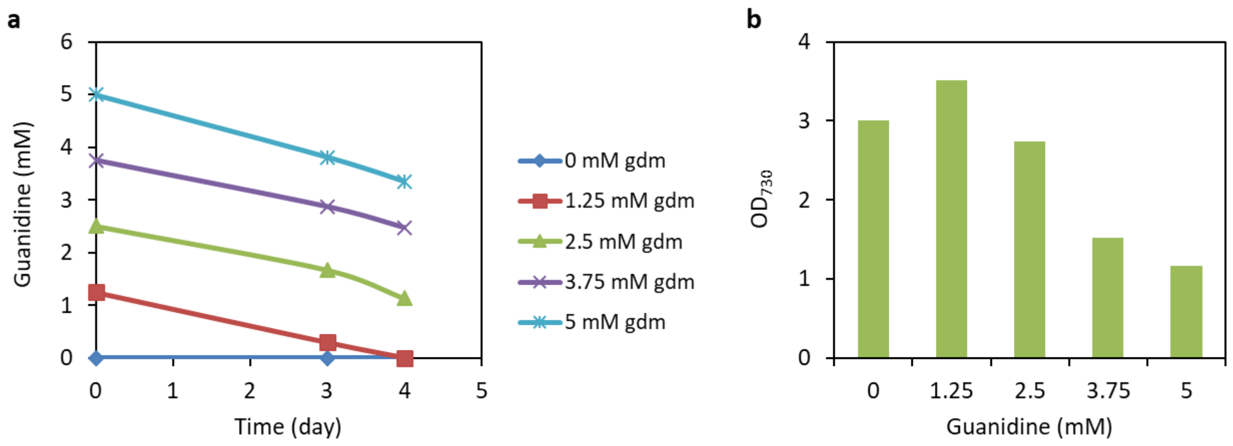

Supplementary Fig. 1. **Degradation of guanidine by *Synechocystis* culture with nitrate gradually replaced by guanidine in the medium**. *Synechocystis* 6803 was inoculated with an initial OD_{730} of 0.1 in the mBG11 medium with nitrate adjusted from 17.6 mM to 5 mM (0 mM gdm), and then the nitrate was gradually replaced by the equal molar concentrations of guanidine until the entire 5 mM nitrate was replaced by 5 mM guanidine (5 mM gdm). **a**, Guanidine concentrations in the culture media. **b**, OD_{730} of cell cultures three days after inoculation. Source data are provided in the Source Data file.

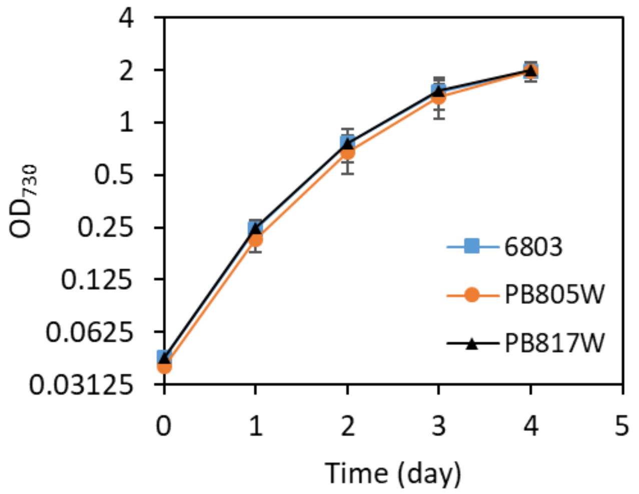

Supplementary Fig. 2. **Comparison of cell growth of Synechocystis strains PCC 6803, PB805W (Δ*sll1077*) and PB817W (*sll1077* overexpressed)**. Cells were grown in BG11 medium supplemented with 10 mM TES-NaOH (pH 8.2) and 50 mM $NaHCO_3$ under constant light of 50 μE m^{-2} s^{-1} on a rotary shaker at 150 rpm and 30 °C. Data represent the means and standard deviations of three independent biological replicates. Source data are provided in the Source Data file.

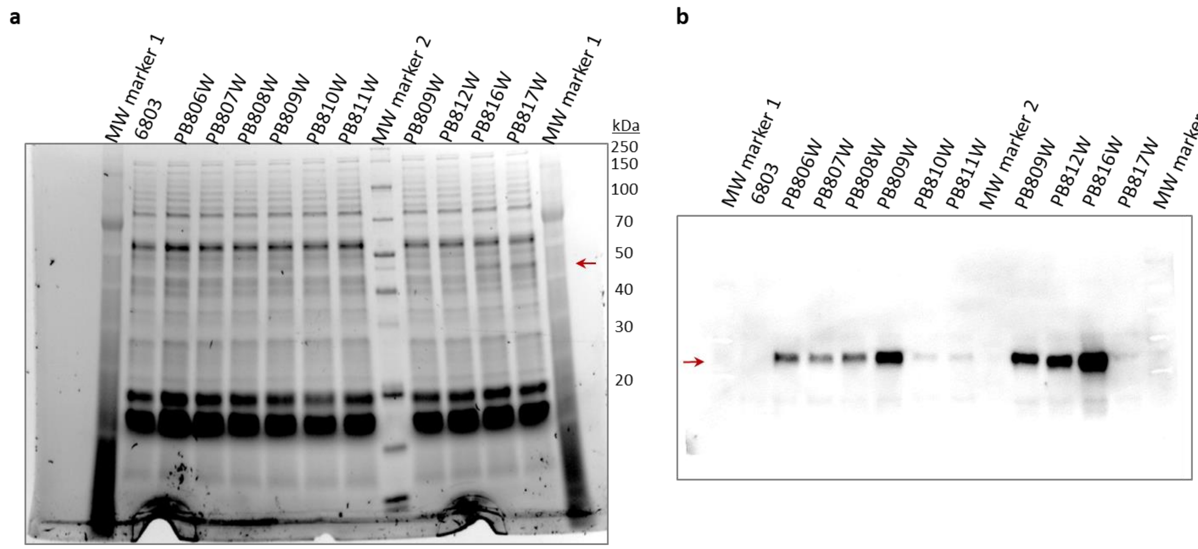

Supplementary Fig. 3. **SDS-PAGE and western blot for comparison of expression levels of sll1077 in recombinant *Synechocystis* strains**. These are an uncropped images for generating Fig. 3b in the main text.

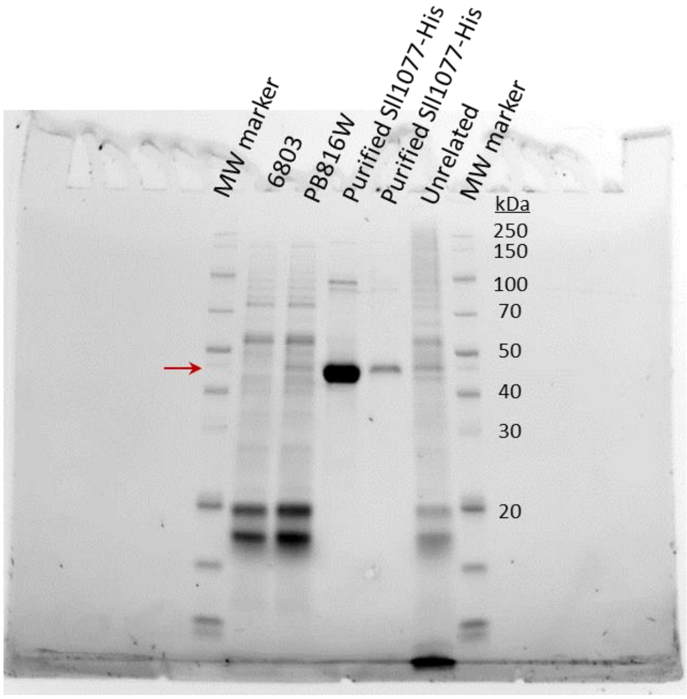

Supplementary Fig. 4. **Uncropped SDS-PAGE image showing the expression of Sll1077-His protein and the purified Sll1077-His protein**. This is an uncropped image that was used for generating Fig. 4a in the main text.

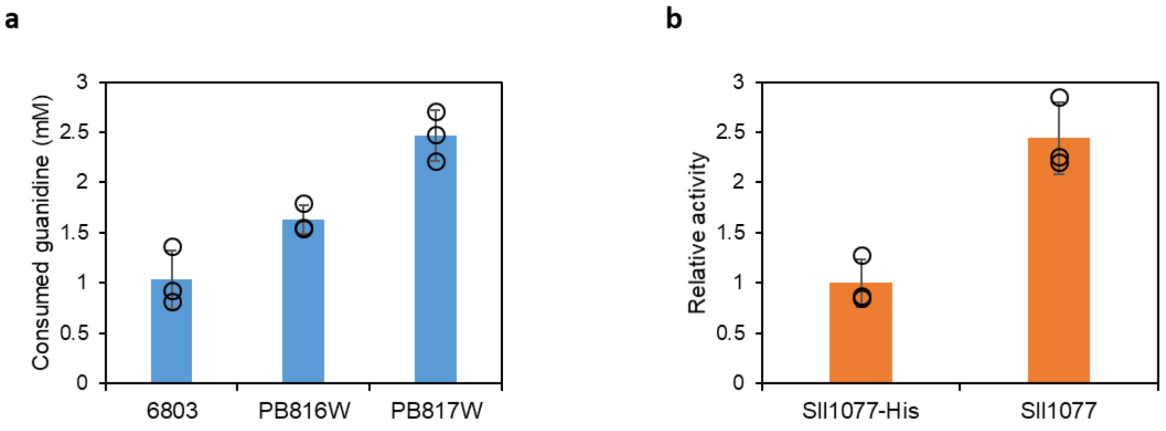

Supplementary Fig. 5. Effect of removal of His-tag on the enzyme activity of Sll1077. Cell lysate containing the same amount of total soluble protein from each strain was incubated with 10 mM guanidine, 1 mM $MnCl_2$, and 20 mM Tris·HCl (pH7.5) at 30 ^oC for three hours. Then, the amounts of guanidine that had been consumed in the solution were calculated. '6803' is the wild-type *Synechocystis* 6803 strain that expresses the native Sll1077; 'PB816W' expresses the native Sll1077 and also expresses the His-tagged Sll1077-His through the recombinant expression cassette; 'PB817W' expresses the native Sll1077 and also expresses the tag-free Sll1077 through the recombinant expression cassette. **a**, Consumed guanidine by each cell lysate. **b**, Relative enzyme activity introduced by expressing Sll1077-His and the untagged Sll1077; calculated based on panel a. Data represents means and standard deviations from three replicates. Source data are provided in the Source Data file.

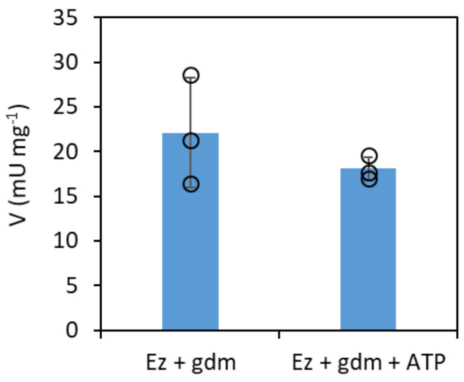

Supplementary Fig. 6. Effect of presence of ATP on the enzyme activity of Sll1077-His. 'Ez + gdm' indicates purified Sll1077-His incubated with 5 mM guanidine, 1 mM $MnCl_2$, 50 mM NaCl and 20 mM Tris·HCl (pH7.5). "Ez + gdm + ATP" is the same as 'Ez + gdm' except that ATP is added to a final concentration of 1 mM. Data represents means and standard deviations from three replicates. Source data are provided in the Source Data file.

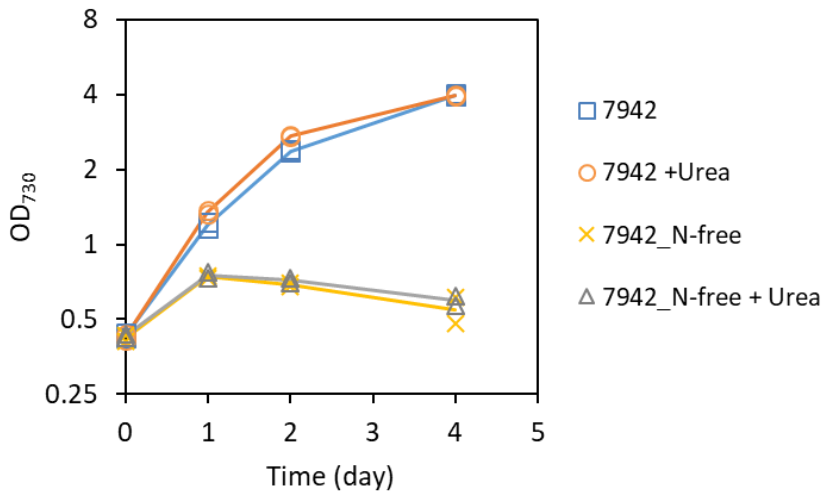

Supplementary Fig. 7. **Tolerance of *Synechococcus* 7942 to urea**. Cell growth curves of *Synechococcus* 7942 grown in nitrate-containing or nitrate-deprived BG11 medium (N-free) supplemented with 50 mM $NaHCO_3$, with or without 5 mM urea, under constant light of 50 μE m^{-2} s^{-1} on a rotary shaker at 150 rpm and 30 °C. Data represent the average of two independent biological replicates and are overlaid with individual data points. Source data are provided in the Source Data file.

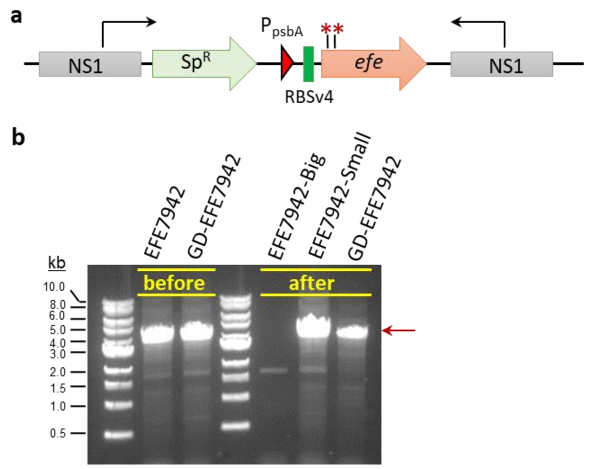

Supplementary Fig. 8. Improved genomic stability of the *efe*-expressing *Synechococcus* 7942 derivative strain through co-overexpressing *sll1077*. **a**, Schematic structure of the EFE expression cassette inserted into the genomes of *Synechococcus elongatus* strains EFE7942 and GD-EFE7942. Arrows indicate the primers used for PCR verification of genotypes of the *efe*-expressing strains. Red asterisks indicate sites where mutation occurred causing early termination of the translation of EFE in the randomly chosen big colonies of strain EFE7942 pictured in Fig. 6a. **b**, Colony PCR results showing the genotypes of *efe*-expressing cells initially grown on agar plates placed at 35 °C (“before”) and then on agar plates placed at 30 °C (“after” as depicted in Fig. 6a). DNA fragments were amplified using primers illustrated as black arrows in a. Red arrow indicates the expected size of the PCR product should cells maintain the correct EFE expression cassette. Data represent results from at least three independent experiments.

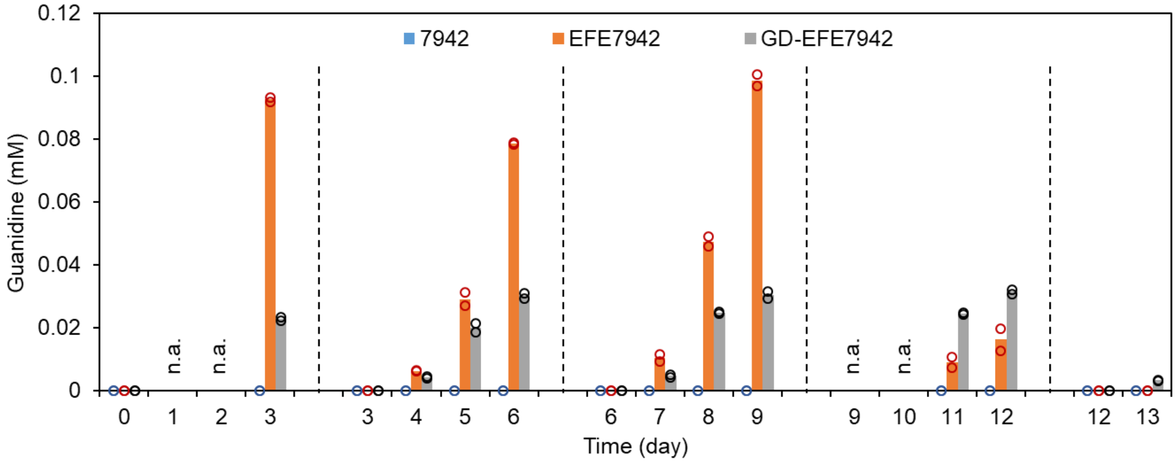

Supplementary Fig. 9. Guanidine accumulative titers in the culture supernatants of *Synechococcus* strains. *Synechococcus* strains 7942, EFE7942 and GD-EFE7942 were grown under the same conditions as in Fig. 6b-d. “n.a.” means samples were not saved for analysis of guanidine. Batch #1, day 0 – 3; batch #2, day 3 – 6; batch #3, day 6 – 9; batch #4, day 9 – 12; batch #5, day 12 – 13. Data represent means (as indicated by the bar chart) of two independent biological replicates and are overlaid with individual data points (sown as empty circles). Source data are provided in the Source Data file.

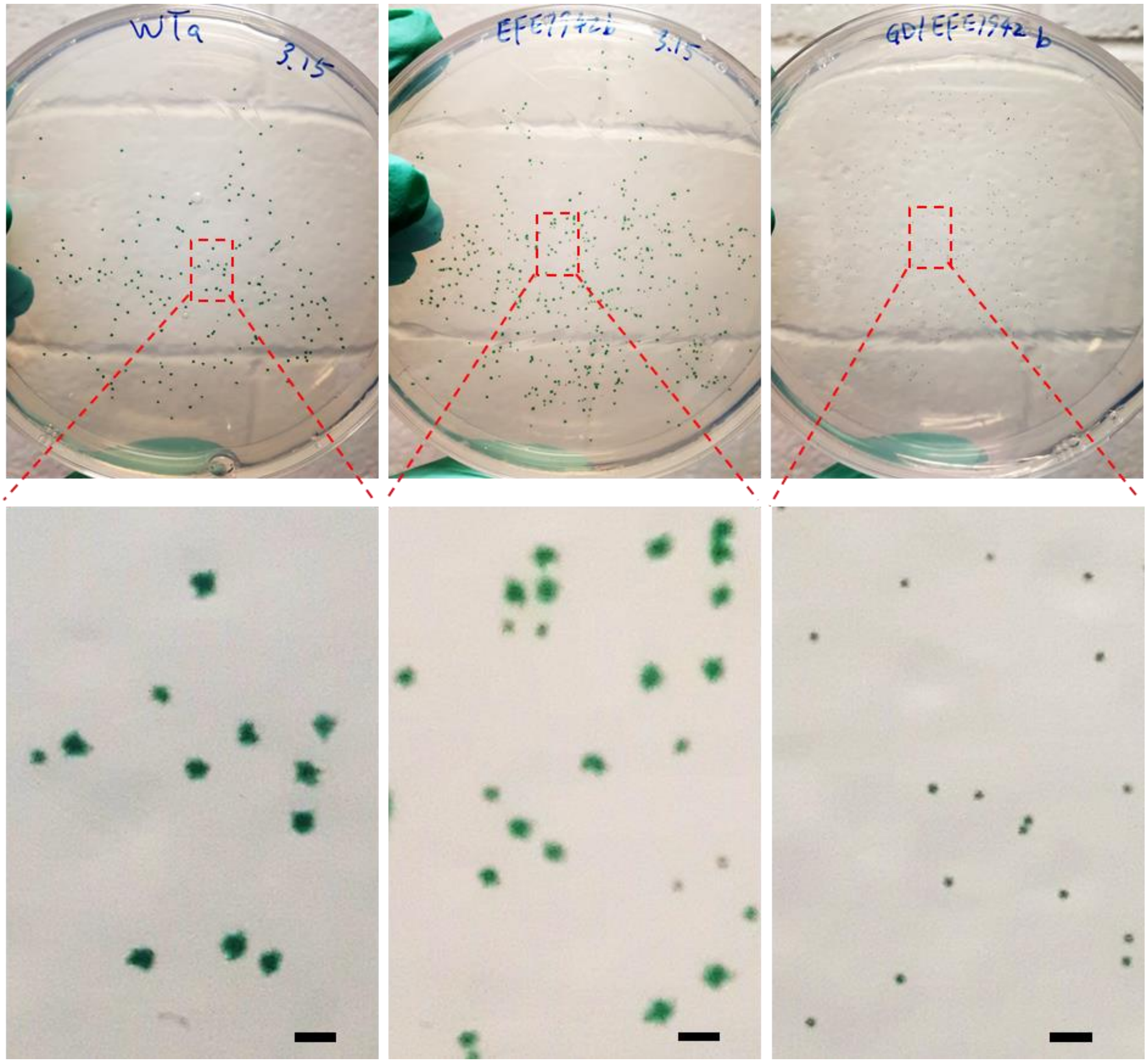

Supplementary Fig. 10. Colonies formed on agar plates spread with diluted 13th day cultures in Fig. 6b-d. *Synechococcus* strains 7942, EFE7942 and GD-EFE7942 were grown under the same conditions as in Fig. 6b-d were diluted and spread on to BG11 agar plates and incubated under light of ~15 μE m^{-2} s^{-1} at ambient temperature for two weeks. Scale bars indicate 1 mm.

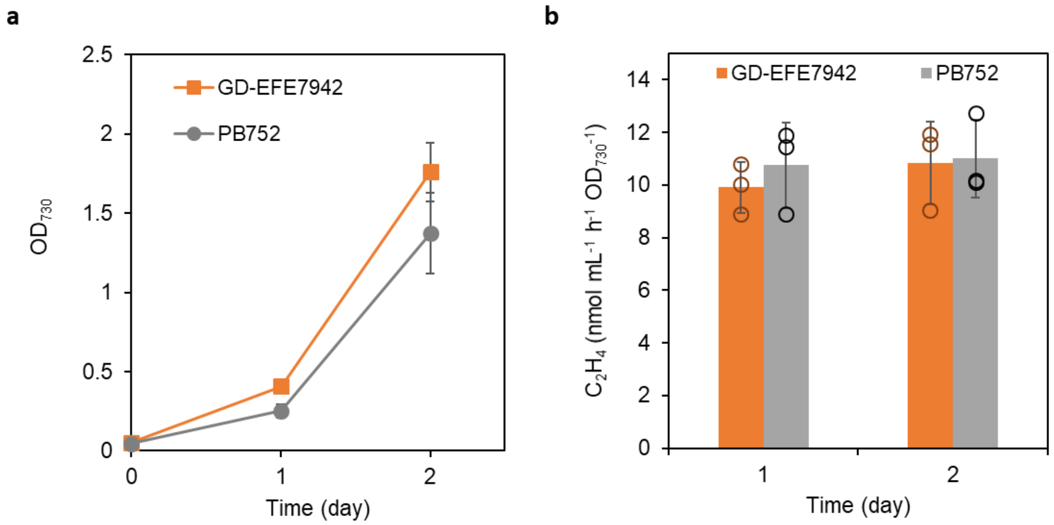

Supplementary Fig. 11. Comparison of ethylene productivities between *efe*-expressing *Synechococcus* strain GD-EFE7942 and *Synechocystis strain* PB752. **a**, Growth of two cyanobacterial strains. **b**, Specific ethylene productivities. Strains were inoculated in the mBG11 medium with an initial OD_{730} of about 0.05, under 120 μE m^{-2} s^{-1}, 130 rpm, 30 °C, aerated with 1% CO_2. Data represents means and standard deviations from three independent biological replicates. Source data are provided in the Source Data file.

```
Sll1077 1   MSDATPFRPPSEAEEALIKETRLPLTGWQQEVDQGLTYGLEAAASIKDRSIPTFSRGELPHYAGINTFMKAP--YLED-- 76
Sll0228 1   -------------------------------------------------------------MHSPNKFTSGPKQFLESEA 19

Sll1077 77  VREVGKYDVAIVGVPHDSGTTYRPGTRFGPQGIRRISALYTPYNFEMGVDLREQISLCDVGDIFTIPANNEKSFD----- 151
Sll0228 20  ITSYADAAVVVVPIPYEATTSYRKGCEHGPEAVLEASDQLEAYDEELGTSPCH-----DLGIYTCAPLADSNKHPALAGD 94

Sll1077 152 ----QISKGIAHIFSSGAFPIILGGDHSIGFPTVRGICRHLGDKKVGIIHFDRHVDTQETDLDERMHTCPWFHATNMANA 227
Sll0228 95  AMVTEVCDGIAPFVEDGKFVVAIGGEHAITTGVVRAMQRGTS-EPFTVVQIDAHGD-MRDKFEGSCHNHACVMRRVLELG 172

Sll1077 228 PAKNLVQLGIGGWQVPRQGVKVCRERATNILTVTDITEMSLDAAADFAIARATDGTDCVWISFDIDCIDAGFVPGTGWPE 307
Sll0228 173 ----LPTLPIAIRAICQEEADLIREKNIPVFWAREMADN--PNWINEAIASIT--TQKVFLTIDMDGFDPGFMPGVGTPE 244

Sll1077 308 PGGLLPREALYLLKRIIRETNVCGMEVVEVSPPYDISDMTSLMATRVICDTMAHLVVSGQLPRTEKPAYIHAEANMAVDE 387
Sll0228 245 PGGLGWYEGLNFFRRLFQTKQVIGCDLMELAPVRG-SVVSEFSTAKLAYKLMGYW---GESQRKKL-------------- 306

Sll1077 388 PWQ 390
Sll0228 ---
```

Supplementary Fig. 12. Comparison of protein sequences of Sll1077 and Sll0228 of the *Synechocystis* 6803 strain. Red letters indicate identical amino acids. Bold letters indicate the conservative putative active cite of agmatinase family proteins[1]. Underscored letters indicate the conservative manganese [Mn] ion binding site[1].

Supplementary Table 1. **All strains and plasmids used in this study.**

Strains	Genotype or features	Sources
NEB5α	*fhuA2 Δ(argF-lacZ)U169 phoA glnV44 Φ80 Δ(lacZ)M15 gyrA96 recA1 relA1 endA1 thi-1 hsdR17*	NEB
Synechocystis sp. PCC 6803	Wild-type	ATCC
Synechocystis PB805W	*Δsll1077* in *Synechocystis* sp. PCC 6803	This study
Synechocystis PB806W	Overexpression (OE) of *slr1077-His* in *Synechocystis* sp. PCC 6803; inserted between *slr1495* and *sll1397*	This study
Synechocystis PB807W	OE of *sll1077-His* in *Synechocystis* sp. PCC 6803	This study
Synechocystis PB808W	OE of *sll1077-His* in *Synechocystis* sp. PCC 6803	This study
Synechocystis PB809W	OE of *sll1077-His* in *Synechocystis* sp. PCC 6803	This study
Synechocystis PB810W	OE of *sll1077-His* in *Synechocystis* sp. PCC 6803	This study
Synechocystis PB811W	OE of *sll1077-His* in *Synechocystis* sp. PCC 6803	This study
Synechocystis PB812W	OE of *sll1077-His* in *Synechocystis* sp. PCC 6803	This study
Synechocystis PB816W	OE of *sll1077-His* in *Synechocystis* sp. PCC 6803	This study
Synechocystis PB817W	OE of *sll1077-His* in *Synechocystis* sp. PCC 6803	This study
Synechocystis PB752	OE of *efe* in *Synechocystis* sp. PCC 6803; inserted at the *slr0168* neutral site	[2]
Synechococcus elongatus PCC 7942	Wild-type	In Lab
Synechococcus elongatus EFE7942	OE of *efe* in *Synechococcus elongatus* PCC 7942; inserted at neutral site 1, *i.e.*, "Synpcc7942_2498"	This study
Synechococcus elongatus GD44	OE of *sll1077* in *Synechococcus elongatus* PCC 7942; inserted at neutral site 4, *i.e.*, “Synpcc7942_0103”	This study
Synechococcus elongatus GD-EFE7942	OE of *sll1077* and *efe* in *Synechococcus elongatus* PCC 7942	This study
**Plasmids**		
pBluescript II SK (+)	Amp^R, pUC ori	Stratagene
pPB305	*sll1077U*-Cm^R-*sll1077D*, inserted to the pBluescript II SK (+) vector backbone	This study
pPB306	*slr1495*-Cm^R-P_{tac}-RBSv306-*sll1077-His-sll1397*, inserted to the pBluescript II SK (+) vector backbone	This study
pPB307	Derivative of pPB306; RBSv306 replaced by RBSv307	This study
pPB308	Derivative of pPB306; RBSv307 replaced by RBSv308	This study
pPB309	Derivative of pPB306; RBSv306 replaced by RBSv309	This study
pPB310	Derivative of pPB306; RBSv306 replaced by RBSv310	This study
pPB311	Derivative of pPB306; RBSv306 replaced by RBSv311	This study
pPB312	Derivative of pPB309; XhoI site between *sll1077* and His tag deleted	This study
pPB316	Derivative of pPB312; *rrnB* terminator added downstream of *sll1077-His*	This study
pPB317	Derivative of pPB316; His tag removed from downstream of *sll1077*	This study
pJU158	*slr0168*-P_{psbA}-RBSv4-*efe*-T_{T7} -Sm^R-*slr0168*, pUC ori	[3]
pEFE-FLAG-NS1	NS1Up- Sm^R-P_{psbA}-RBSv4-*efe*-T_{t7}- NS1Dn	This study
pCX0104-LuxAB-FT	Zn^{++}-inducible expression of P_{smtA}::*luxAB::*3×FLAG targeted to NS4 (Cm^R)	[4]
pGD6803-NS4	NS4Dn-P_{tac}-RBSv309-*sll1077*-T_{rrnB}-Cm^R-NS4Up	This study

Supplementary Table 2 | Primers used in this study.

Plasmids	Primers	DNA sequences	Target	Template source
pPB305	Sll1077U3	GGGCGAATTGGGTACCggagtttcggttaagtctaag (KpnI)	Sll1077U	S6803 gDNA
	Sll1077U4	CACAGGTATCTGCAGaggtagttactagctaaacaac	Sll1077U	S6803 gDNA
	Sll1077D3	GAAGCAGTGTGGGATCCTagtaactttaacttgactaattattgc	Sll1077D	S6803 gDNA
	Sll1077D4	GAACAAAAGCTGGAGCTCcgagcagaacagttttacc (SacI)	Sll1077D	S6803 gDNA
	CatU3	gtaactacctCTGCAGATACCTGTGACGGAAGATCAC	cat	pACYC184
	CatD4	aaagttactAGGATCCCACACTGCTTCCGGTAGTC	cat	pACYC184
pPB300	sll1077U1	agatataCATATGagcgatgccaccccgtttc	sll1077	S6803 gDNA
	sll1077D2	ggtgCTCGAGttgccagggctcatccactg	sll1077	S6803 gDNA
pPB306	sll1077U3	CAATTTCACACAAggaggatataCATATGagcgatgccacccc gtttc	sll1077	pPB300
	sll1077-His-D4	caattcgcactgaatctccaGTCGACGTTAGCAGCCGGATCTT AGTG	sll1077	pPB300
pPB307	TACR4	GTGTGAAATTGTTATCCGCTCAC	RBS of pPB306d	pPB306d
	sll1077U307	AAGGAGGAAACATatgagcgatgccaccccgtttc	RBS of pPB306d	pPB306d
pPB308	TACR4	GTGTGAAATTGTTATCCGCTCAC	RBS of pPB306d	pPB306d
	sll1077U308	AAGGAGGAACAGCatgagcgatgccaccccgtttc	RBS of pPB306d	pPB306d
pPB309	TACR4	GTGTGAAATTGTTATCCGCTCAC	RBS of pPB306d	pPB306d
	sll1077U309	AAGGAGAAACAGCatgagcgatgccaccccgtttc	RBS of pPB306d	pPB306d
pPB310	TACR4	GTGTGAAATTGTTATCCGCTCAC	RBS of pPB306d	pPB306d
	sll1077U310	AAGAAGGAGAAACAGCatgagcgatgccaccccgtttc	RBS of pPB306d	pPB306d
pPB311	TACR4	GTGTGAAATTGTTATCCGCTCAC	RBS of pPB306d	pPB306d
	sll1077U311	AAGAAGGAGAAACATAGCatgagcgatgccaccccgtttc	RBS of pPB306d	pPB306d
pPB312	sll1077C-F-3xHis	CACCACCACTAAGATCCGGCTG	sll1077- His	pPB309
	sll1077C-R-3xHis	GTGGTGGTGttgccagggctcatccactg	sll1077- His	pPB309
pPB313	sll1077C-F-TAA	TAAGATCCGGCTGCTAAC	sll1077	pPB309
	sll1077C-R	TTAttgccagggctcatc	sll1077	pPB309
pPB316	Primer rrnBU-sll1077	TAAGATCCGGCTGCTAACAAGCTTGCCTGATACA GATTAAATCAGAAC	*rrnBT1T2*	*E. coli* NEB5α gDNA
	Primer rrnBD-SL1	caattcgcactgaatctccaGTCGACcaggaagagtttgtagaaacg	*rrnBT1T2*	*E. coli* NEB5α gDNA
pPB317	Primer rrnBU-sll1077	TAAGATCCGGCTGCTAACAAGCTTGCCTGATACA GATTAAATCAGAAC	*rrnBT1T2*	*E. coli* NEB5α gDNA
	Primer rrnBD-SL1	caattcgcactgaatctccaGTCGACcaggaagagtttgtagaaacg	*rrnBT1T2*	*E. coli* NEB5α gDNA

pEFE-FLAG-NS1		Blunt end cloning; no primers used		pJU158
pGD7942-NS4		Blunt end cloning; no primers used		pPB317
Following primers were used to verify mutation at the *slr0168* neutral site of genome of *Synechocystis* 6803				
	US168e1	CAAGAGTAGTTCCCTCAACAC		
	US168e2	CTGAAGGGATTACGCAATAC		
Following primers were used to verify the mutation at the *slr1495-sll1397* neutral site of genome of *Synechocystis* 6803				
	VF1a	GTC TCC AGG ATG CGT TAA C		
	VR1a	CGA TGC AAG ATT GAT AGA CAG AG		
Following primers were used to verify the mutation at the *sll1077* site of genome of *Synechocystis* 6803				
	Sll1077e1	ggcaattgttgattgagttg		
	Sll1077e2	gaggtgaatcttggtgatttg		
Following primers were used to verify the mutation at the neutral site 1, *i.e.*, "Synpcc7942_2498" site, of genome of *S. elongatus* PCC 7942				
	NS13	GTGCAGCAGCAACTTCAAG		
	NS14	GTGCGTTCCACAGACATC		
	NS15	GGCTGCTTGGCAAAAAC		
	NS16	CCTGTTGTGCTGTTTCGATTG		
Following primers were used to verify the mutation at neutral site 4, *i.e.*, “Synpcc7942_0103” site, of genome of *S. elongatus* PCC 7942				
	5’NS4	tcttgctctgacgccttattc		
	3’NS4	atcgtcccaagatccagaatgt		

Supplementary References

1. Sekowska, A.; Danchin, A.; Risler, J. L., Phylogeny of related functions: the case of polyamine biosynthetic enzymes. *Microbiology (Reading)* **2000,** *146 (Pt 8)*, 1815-1828.
2. Wang, B.; Eckert, C.; Maness, P. C.; Yu, J., A Genetic Toolbox for Modulating the Expression of Heterologous Genes in the Cyanobacterium Synechocystis sp. PCC 6803. *ACS Synth Biol* **2018,** *7* (1), 276-286.
3. Xiong, W.; Morgan, J. A.; Ungerer, J.; Wang, B.; Maness, P.-C.; Yu, J., The plasticity of cyanobacterial metabolism supports direct CO2 conversion to ethylene. *Nature Plants* **2015,** *1*, 15053.
4. Cheah, Y. E.; Xu, Y.; Sacco, S. A.; Babele, P. K.; Zheng, A. O.; Johnson, C. H.; Young, J. D., Systematic identification and elimination of flux bottlenecks in the aldehyde production pathway of Synechococcus elongatus PCC 7942. *Metab. Eng.* **2020,** *60,* 56-65.
