# Supplementary material for: A guanidine-degrading enzyme controls genomic stability of ethylene-producing cyanobacteria
Source: Nat Commun. 2021 Aug 26;12:5150. doi: 10.1038/s41467-021-25369-x (PMC8390497; doi:10.1038/s41467-021-25369-x)
Supplement: Supplementary file 8 — Description of additional supplementary files [file 41467_2021_25369_MOESM8_ESM.docx]

Description of additional supplementary information

Title: Supplementary Data 1

Description: Comparative proteomics for Synechocystis PCC 6803 and JU547.

Title: Supplementary Data 2

Description: Intact and spontaneously mutated efe-expression cassette on Synechococcus genome.

Title: Supplementary Data 3

Description: Annotated “arginases” under the control of guanidine riboswitches.

Title: Supplementary Data 4

Description: Top 1000 hits of BLAST analysis of Protein Sequence of Sll1077.

Title: Supplementary Data 5

Description: DNA sequences of representative recombinant DNA constructs and plasmids
